# Supplementary material for: Investigating cone photoreceptor development using patient-derived NRL null retinal organoids
Source: Commun Biol. 2020 Feb 21;3:82. doi: 10.1038/s42003-020-0808-5 (PMC7035245; doi:10.1038/s42003-020-0808-5)
Supplement: Supplementary file 1 — Reporting Summary [file 42003_2020_808_MOESM1_ESM.pdf]

nature research

Corresponding author(s): Donald Zack and David Gamm

Last updated by author(s): 1/14/2020

Reporting Summary

Nature Research wishes to improve the reproducibility of the work that we publish. This form provides structure for consistency and transparency in reporting. For further information on Nature Research policies, see Authors & Referees and the Editorial Policy Checklist.

Statistics

For all statistical analyses, confirm that the following items are present in the figure legend, table legend, main text, or Methods section.

n/a	Confirmed	
☐	☒	The exact sample size (*n*) for each experimental group/condition, given as a discrete number and unit of measurement
☐	☒	A statement on whether measurements were taken from distinct samples or whether the same sample was measured repeatedly
☐	☒	The statistical test(s) used AND whether they are one- or two-sided<br>*Only common tests should be described solely by name; describe more complex techniques in the Methods section.*
☒	☐	A description of all covariates tested
☐	☒	A description of any assumptions or corrections, such as tests of normality and adjustment for multiple comparisons
☐	☒	A full description of the statistical parameters including central tendency (e.g. means) or other basic estimates (e.g. regression coefficient) AND variation (e.g. standard deviation) or associated estimates of uncertainty (e.g. confidence intervals)
☐	☒	For null hypothesis testing, the test statistic (e.g. *F*, *t*, *r*) with confidence intervals, effect sizes, degrees of freedom and *P* value noted<br>*Give P values as exact values whenever suitable.*
☒	☐	For Bayesian analysis, information on the choice of priors and Markov chain Monte Carlo settings
☒	☐	For hierarchical and complex designs, identification of the appropriate level for tests and full reporting of outcomes
☒	☐	Estimates of effect sizes (e.g. Cohen's *d*, Pearson's *r*), indicating how they were calculated

Our web collection on statistics for biologists contains articles on many of the points above.

Software and code

Policy information about availability of computer code

Data collection	NA
Data analysis	Seurat R package (version 2), Monocle R package (version 2), dropseq cookbook to extract expression matrices from fastq files (version 1.0.1)

For manuscripts utilizing custom algorithms or software that are central to the research but not yet described in published literature, software must be made available to editors/reviewers. We strongly encourage code deposition in a community repository (e.g. GitHub). See the Nature Research guidelines for submitting code & software for further information.

Data

Policy information about availability of data

All manuscripts must include a data availability statement. This statement should provide the following information, where applicable:

- Accession codes, unique identifiers, or web links for publicly available datasets
- A list of figures that have associated raw data
- A description of any restrictions on data availability

Raw sequencing files and dge expression matrices can be downloaded from GEO, accession number GSE143669

Field-specific reporting

Please select the one below that is the best fit for your research. If you are not sure, read the appropriate sections before making your selection.

☒ Life sciences ☐ Behavioural & social sciences ☐ Ecological, evolutionary & environmental sciences

Life sciences study design

All studies must disclose on these points even when the disclosure is negative.

Sample size	for d100 Dropseq: 2 WT each from 2 WT lines and 2 organoids each of 2 clones of L75Pfs<br>for d170 Dropseq: 3 WT organoids and 3 each of 2 clones of L75Pfs<br>Quantification of photoreceptors populations in stage 3 organoids by histology: 4-5 organoids each from 3 WT lines and 3 L75Pfs clones<br>these sample sizes were chosen to ensure consistency within each genotype and ability to identify any outlier organoids
Data exclusions	no exclusions
Replication	no replication was performed as replicates within each group were consistent with each other
Randomization	not relevant - there were no experimental groups
Blinding	no blinding - samples were not assigned to experimental groups and were rather compared based on genotype

Reporting for specific materials, systems and methods

We require information from authors about some types of materials, experimental systems and methods used in many studies. Here, indicate whether each material, system or method listed is relevant to your study. If you are not sure if a list item applies to your research, read the appropriate section before selecting a response.

Materials & experimental systems

n/a	Involved in the study	
☐	☒	Antibodies
☐	☒	Eukaryotic cell lines
☒	☐	Palaeontology
☒	☐	Animals and other organisms
☐	☒	Human research participants
☒	☐	Clinical data

Methods

n/a	Involved in the study	
☒	☐	ChIP-seq
☒	☐	Flow cytometry
☒	☐	MRI-based neuroimaging

Antibodies

Antibodies used	antibody list available in supplementary table 1
Validation	validation performed using human or monkey retinal sections

Eukaryotic cell lines

Policy information about cell lines

Cell line source(s)	iPSCs
Authentication	PCR and Sanger sequencing to confirm L75Pfs mutation
Mycoplasma contamination	cell lines tested negative for mycoplasma
Commonly misidentified lines (See ICLAC register)	NA

Human research participants

Policy information about studies involving human research participants

Population characteristics	patient with L75Pfs mutation in NRL
Recruitment	the patient was recruited by Dr. Eric Pierce to participates in research on the genetics of inherited retinal diseases
Ethics oversight	University of Wisconsin-Madison and Massachusetts Eye and Ear Infirmary

Note that full information on the approval of the study protocol must also be provided in the manuscript.
