# Supplementary material for: Investigating cone photoreceptor development using patient-derived NRL null retinal organoids
Source: Commun Biol. 2020 Feb 21;3:82. doi: 10.1038/s42003-020-0808-5 (PMC7035245; doi:10.1038/s42003-020-0808-5)
Supplement: Supplementary file 2 — Supplementary Information [file 42003_2020_808_MOESM2_ESM.pdf]

Supplementary Figures

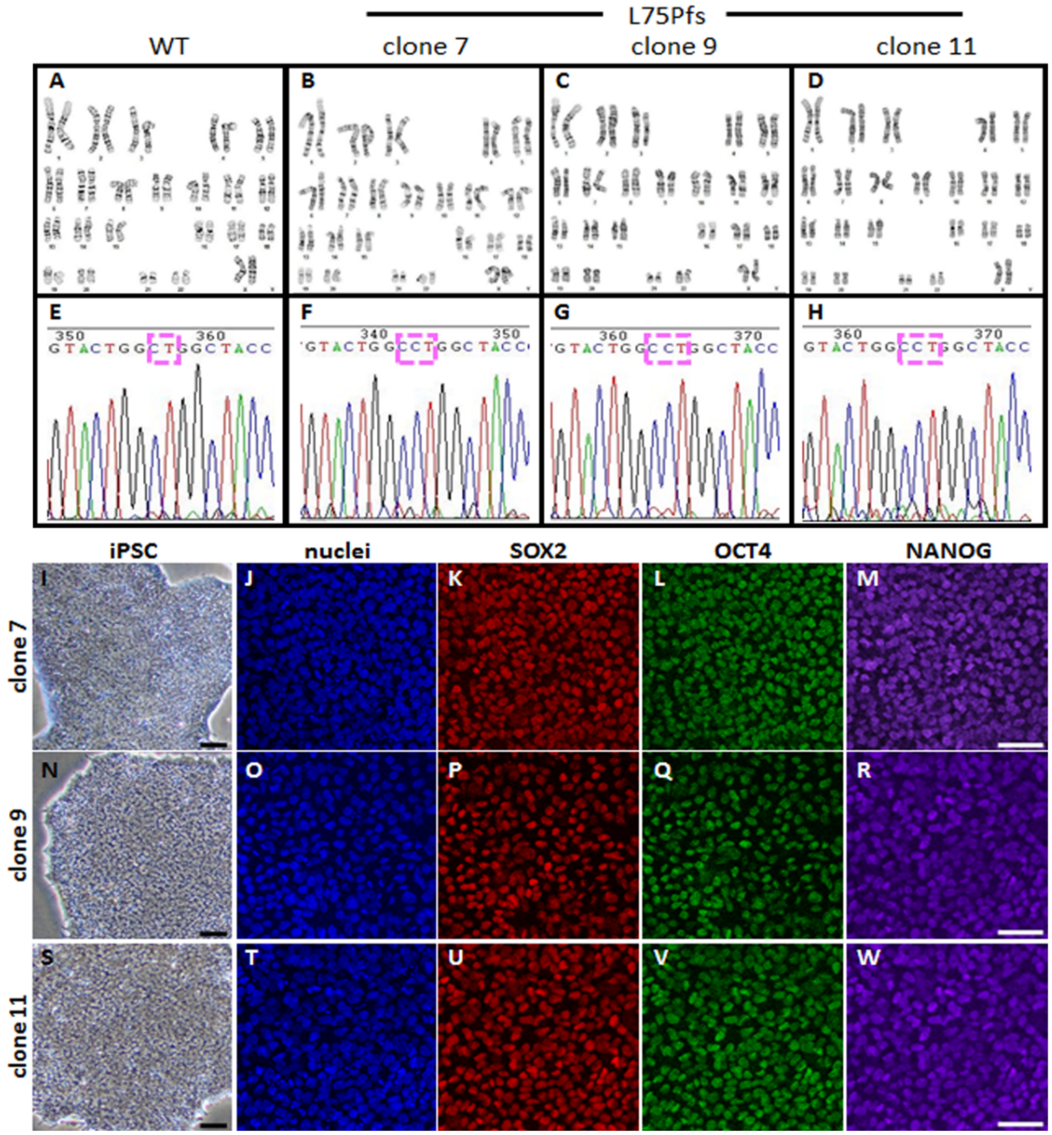

Supplementary Figure 1. Confirmation of normal karyotypes and the presence or absence of *NRL* mutations in the L75Pfs or WT iPSC lines, respectively. A-D) Normal karyotypes were confirmed for hPSCs from WT and 3 independent L75Pfs clones. **E-H)** Sequence of PCR amplicon surrounding *NRL* c.223 from WT iPSCs (**E**) and three L75Pfs clones (**F-H**) (c.233dupC outlined by dotted pink box). **I-W**) Bright field (**I, N, S**) and confocal (**J-M, O-R, T-W**) images of the three L75Pfs iPSC clones confirming the morphology and presence of pluripotency markers. Scale bars = 50 microns.

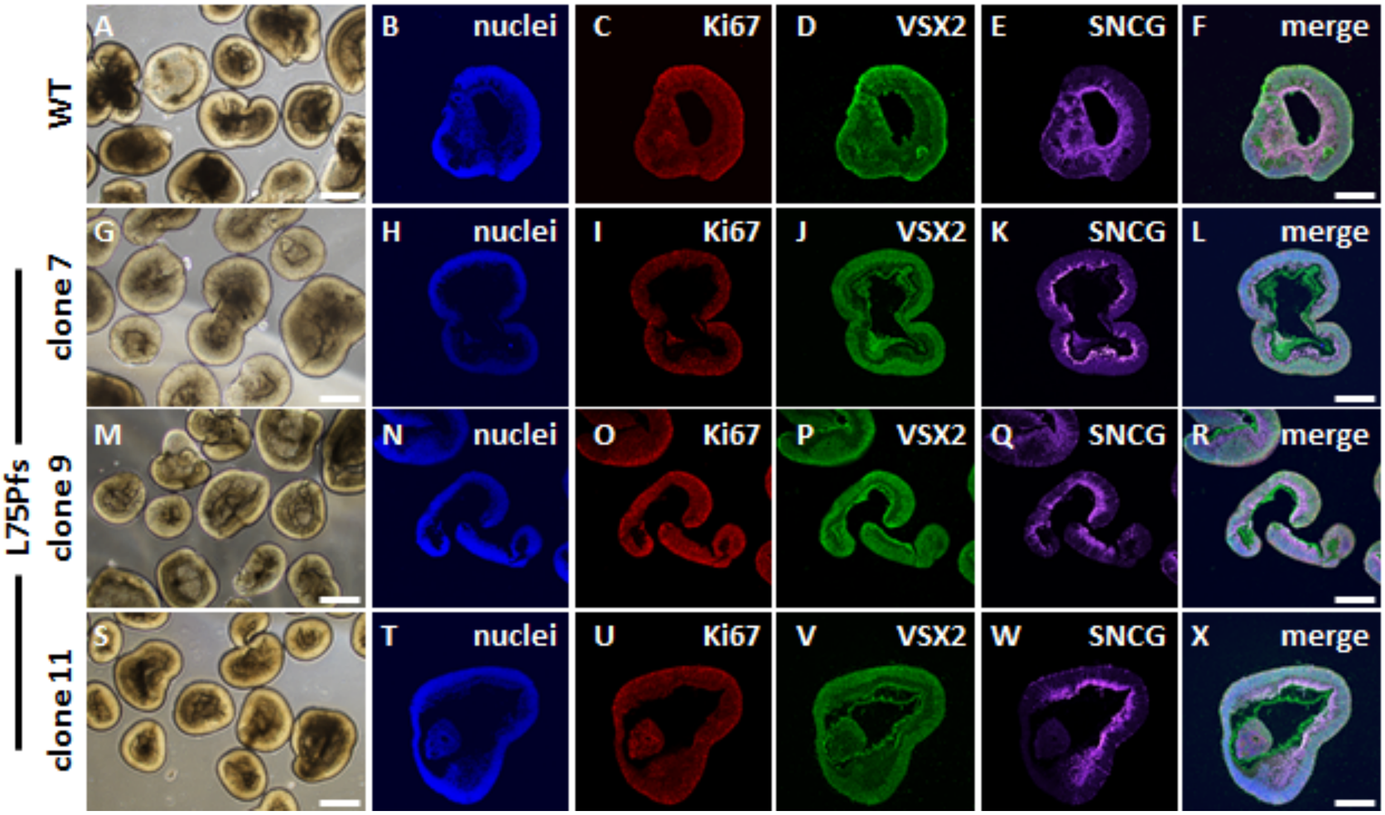

Supplementary Figure 2. All three L75Pfs hiPSC clones display normal early (stage 1) retinal organoid differentiation. Bright field and confocal images of stage 1 retinal organoids demonstrating normal bright field morphology (**A**, **G**, **M**, **S**), as well as the expected presence of proliferative Ki67+/VSX2+ (**C, D**, **I, J**, **O, P**, **U, V**) retinal progenitor cells, and SNCG+ (**E**, **K**, **Q**, **W**) retinal ganglion cells from WT (**A-F**) and L75Pfs organoids from 3 independent lines (**G-X**). Scale bars = 250 microns.

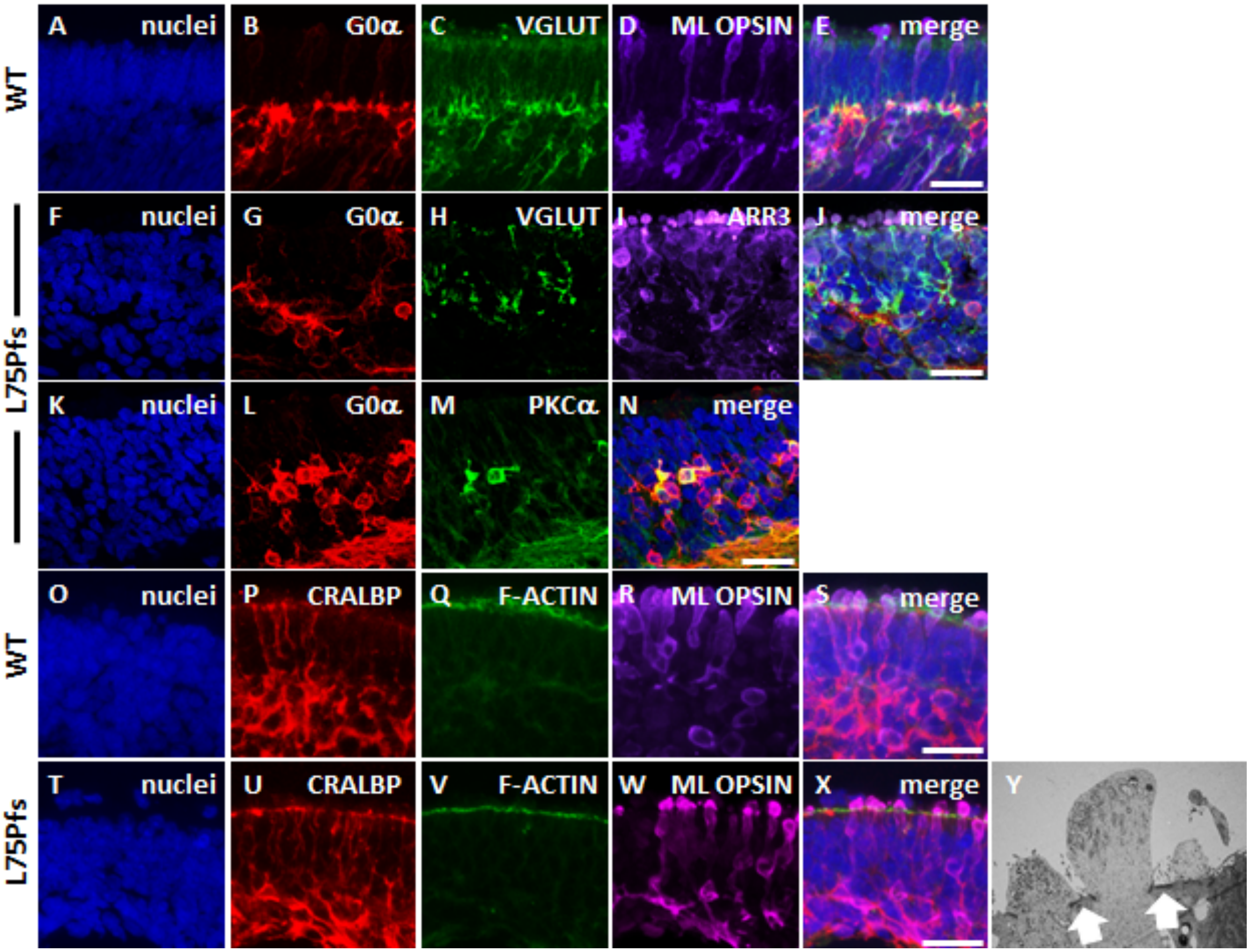

Supplementary Figure 3. The inner nuclear layer (INL), rod ON bipolar cells, and the outer limiting membrane (OLM) develop normally in L75Pfs retinal organoids. A-J) G0α ON bipolar cells (**B, G**) and the presynaptic marker VGLUT (**C, H**) are detected in the INL and outer plexiform-like region (respectively) of WT (**A-E**) and L75Pfs (**F-J)** retinal organoids. **K-N)** G0α+/PKCα + rod ON bipolar cells (**G, H;** merge in **I**) differentiate along with G0α+/PKCα- cone ON bipolar cells in L75Pfs organoids despite the absence of NRL+ rods. **O-X)** F-actin (**Q, V**) and CRALBP+ Müller glia (**P, U**) form an intact OLM in WT (**O-S**) and L75Pfs (**T-X**) retinal organoids (also visualized in L75Pfs by TEM in panel **Y**). Scale bars **A-N** = 25 microns; **Y** = 2 microns.

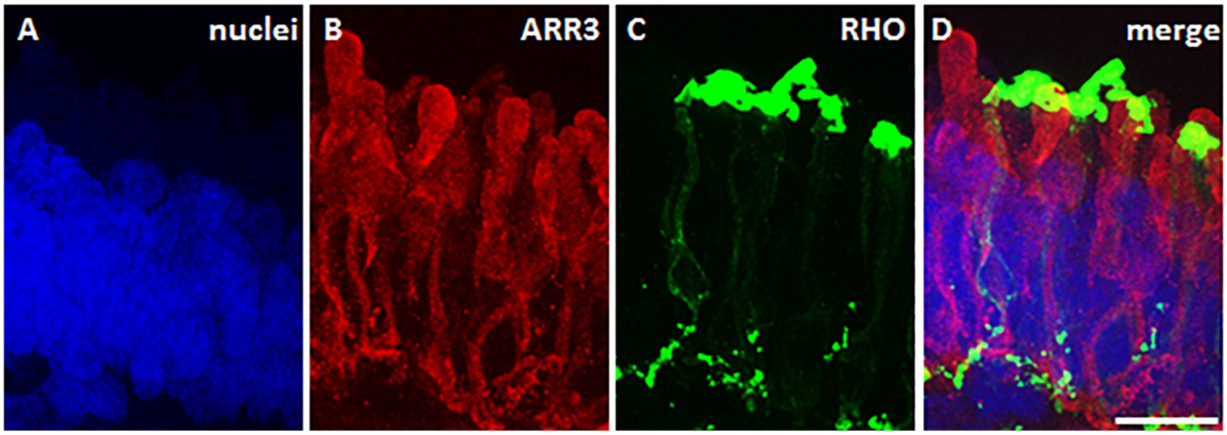

Supplementary Figure 4. Confocal images of ARR3 positive cones (**B**) and RHO positive rods (**C**, merge in **D**; DAPI-stained nuclei are shown in **A**) in a WT d240 stage 3 organoid for comparison to RHO protein expression seen in L75Pfs photoreceptors transduced with lenti-pgkNRL in Figure 3U-X. Scale bar = 25 microns.

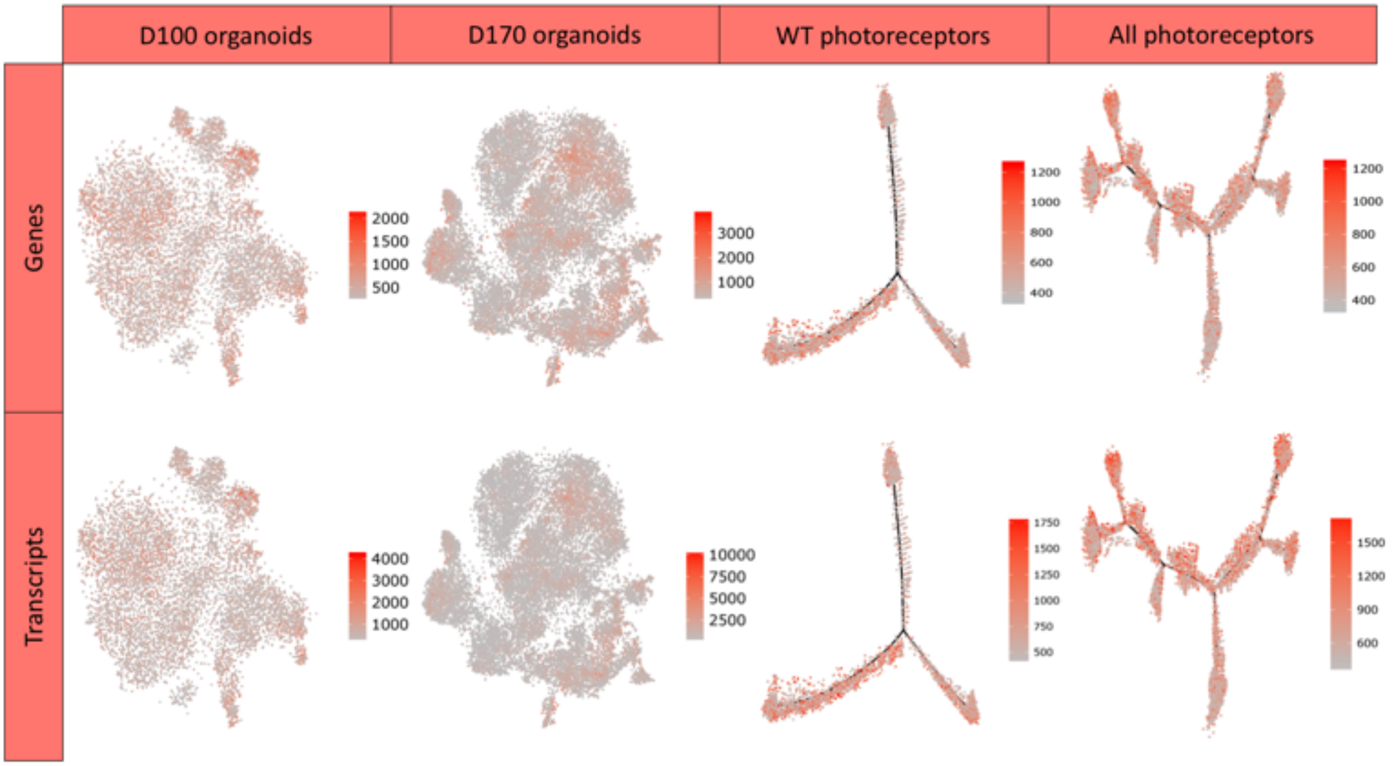

Supplementary Figure 5: UMIs and transcript number per cells mapped onto tSNE plots and pseudotime trajectories.

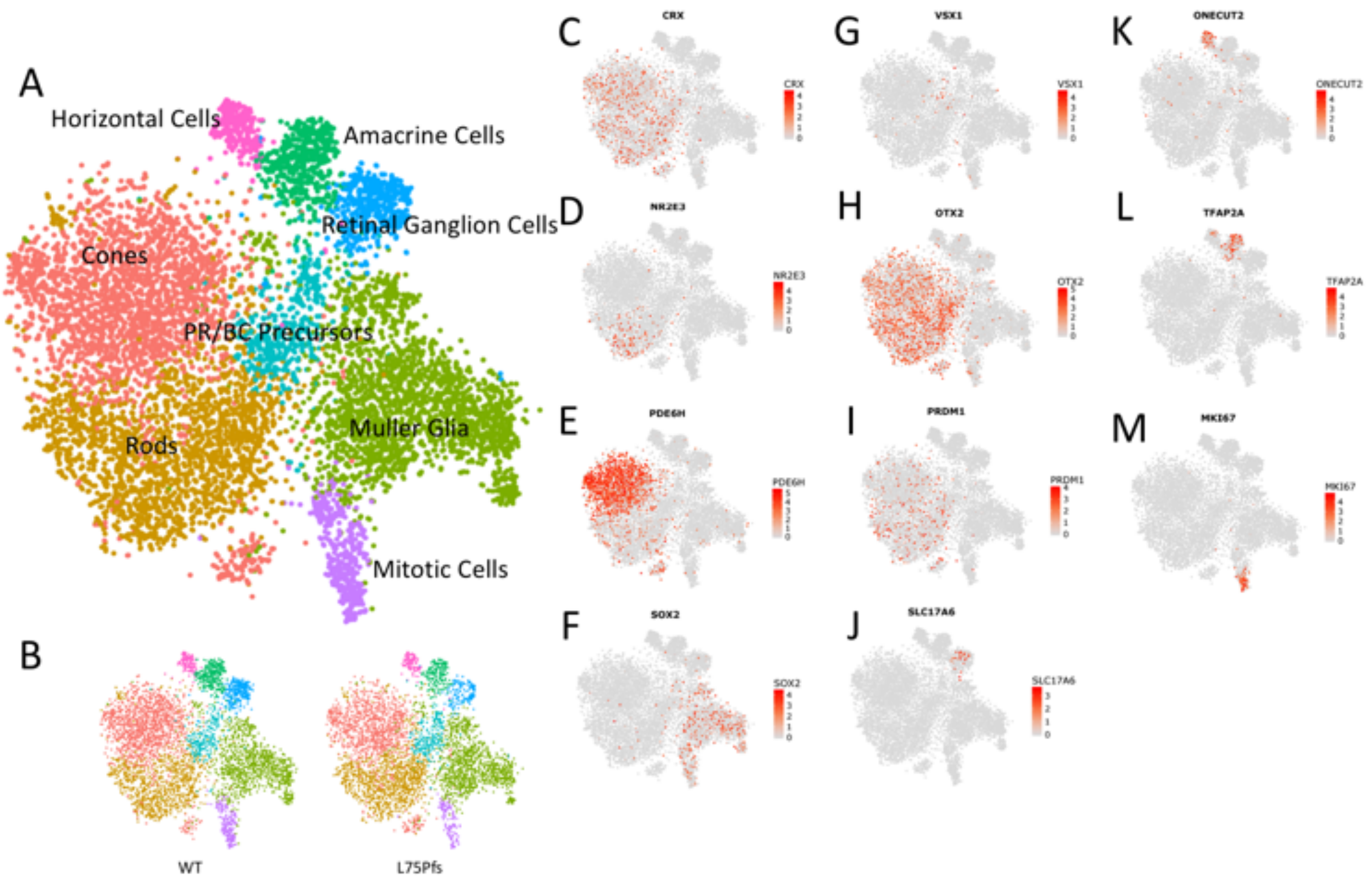

Supplementary Figure 6: Identification of d100 organoid cell populations by marker gene expression. **A)** tSNE plot showing cell populations present in all d100 organoids. **B)** tSNE plot from panel A showing only WT cells (left) or L75Pfs cells (right). **C-M)** Expression pattern of marker genes used to assign cluster identity.

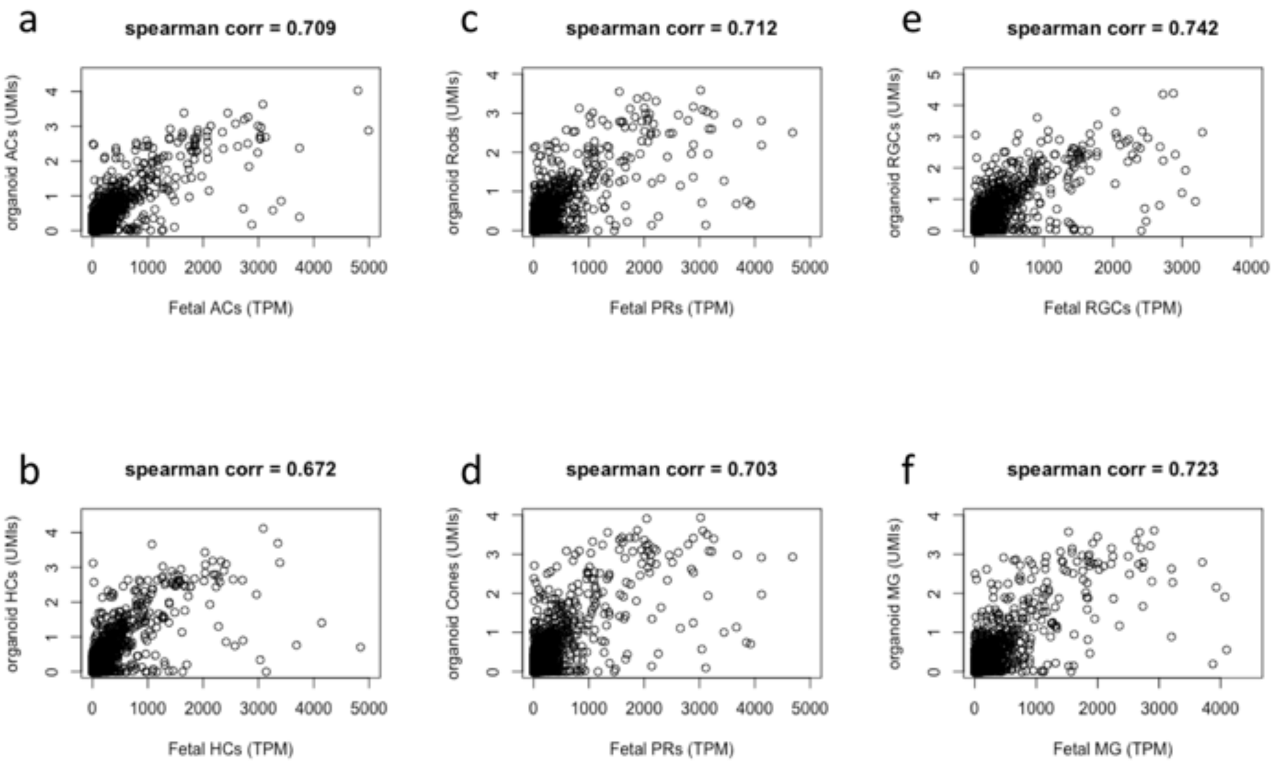

Supplementary Figure 7: Correlation of d100 retinal cell populations with fetal retina scRNAseq data[23]. **A-F)** Scatter plots and spearman correlations of amacrine cells **(A)**, horizontal cells **(B)**, rods **(C)**, cones **(D)**, retinal ganglion cells **(E)**, and Müller glia **(F)**.

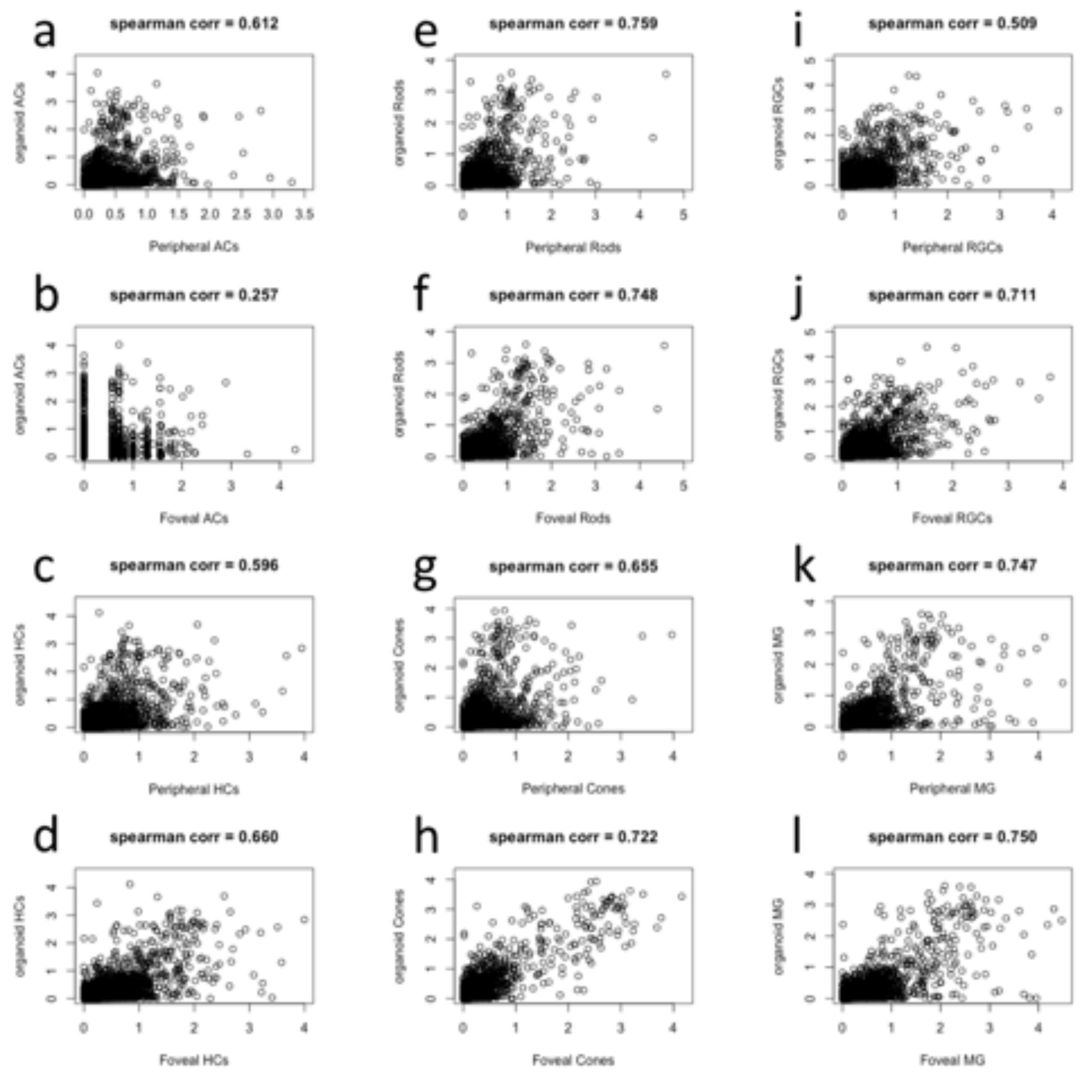

Supplementary Figure 8: Correlation of d100 retinal cell populations with adult peripheral and foveal retina scRNAseq data[24]. **A-L)** Scatter plots and spearman correlations with peripheral **(A)** and foveal **(B)** amacrine cells, peripheral **(C)** and foveal **(D)** horizontal cells, peripheral **(E)** and foveal **(F)** rods, peripheral **(G)** and foveal **(H)** cones, peripheral **(I)** and foveal **(J)** retinal ganglion cells, and peripheral **(K)** and foveal Müller glia **(L)**.

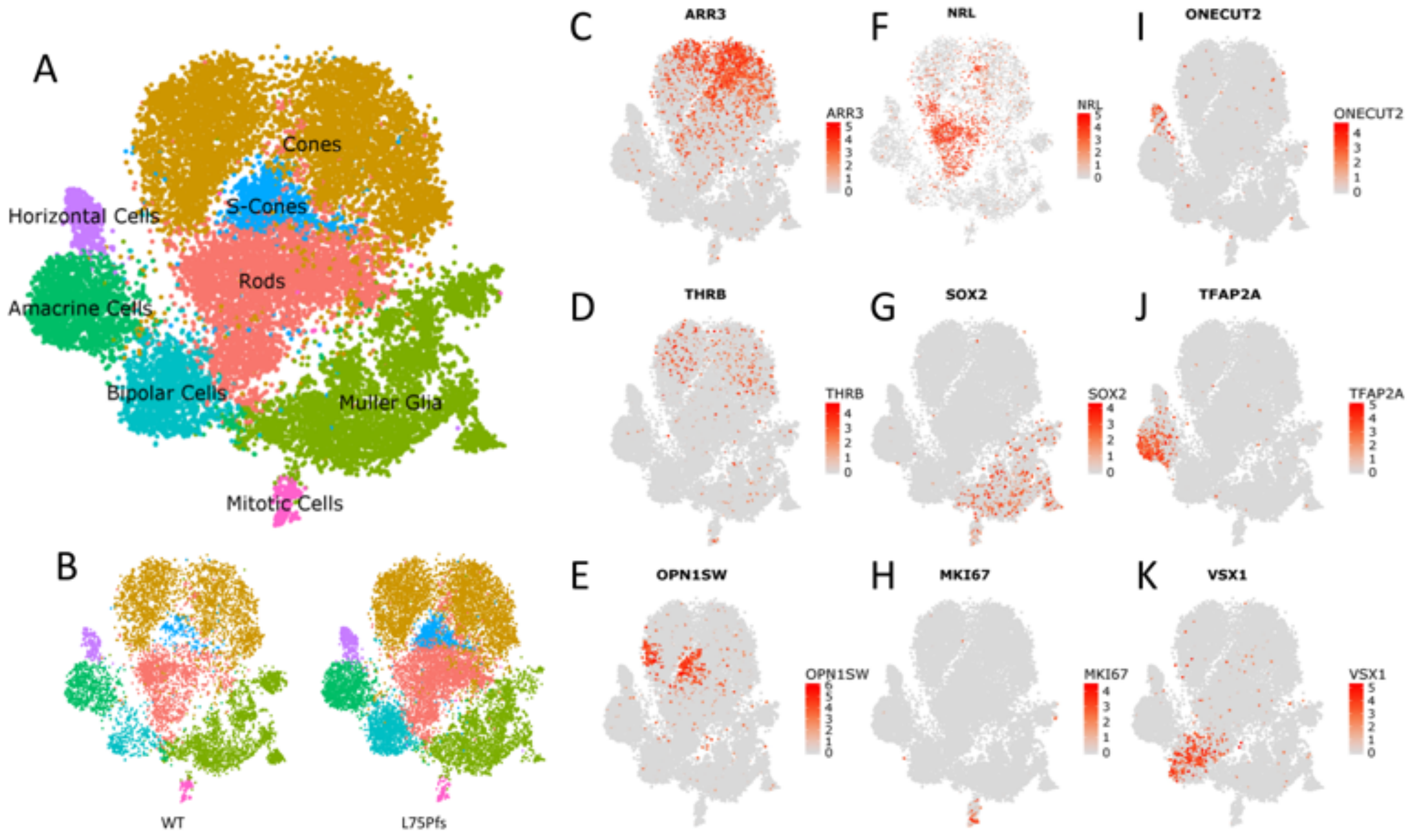

Supplementary Figure 9: Identification of d170 organoid cell populations by marker gene expression.

A) tSNE plot showing cell populations present in all day 170 organoids. **B)** tSNE plot from panel A showing only WT cells (left) or L75Pfs cells (right). **C-K)** Expression patterns of marker genes used to assign cluster identity. For *NRL* expression **(F)**, only WT cells are shown.

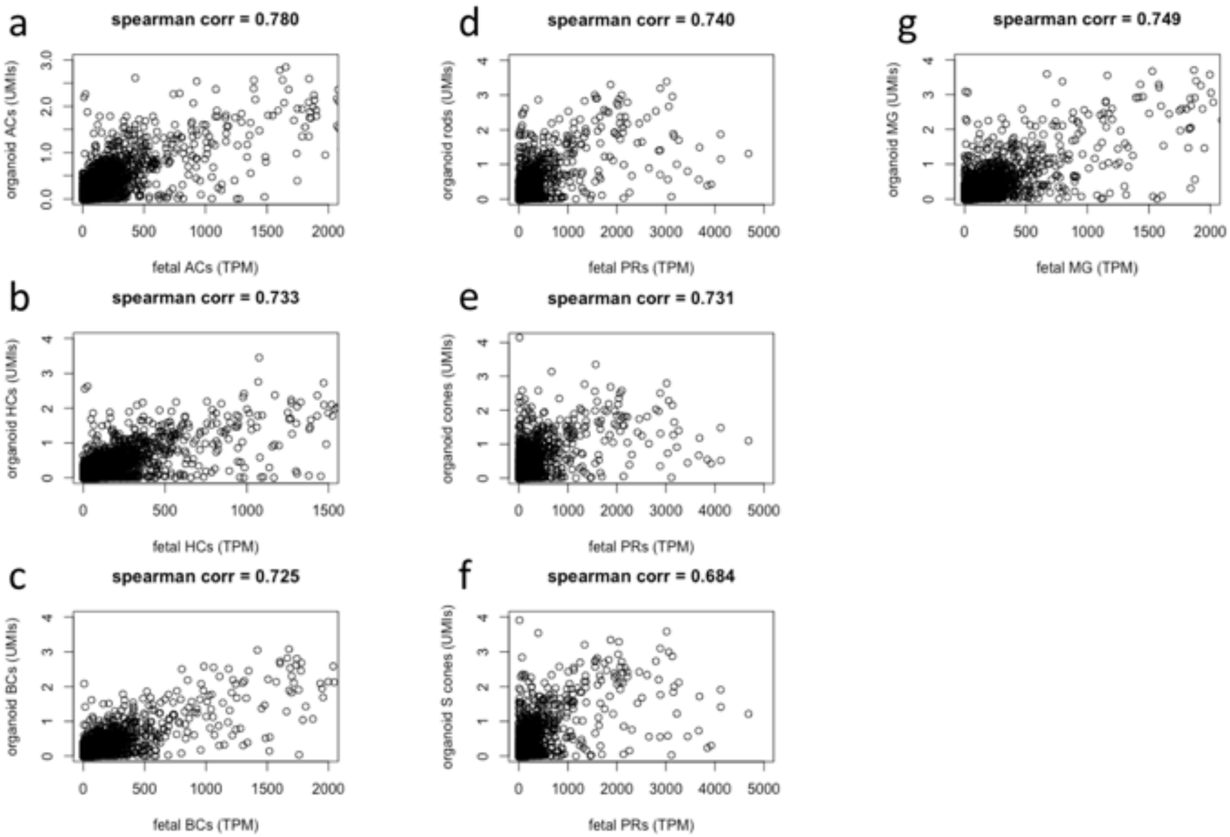

Supplementary Figure 10: Correlation of d170 retinal cell populations with fetal retina scRNAseq data[23]. **A-G)** Scatter plots and Spearman correlations of amacrine cells **(A)**, horizontal cells **(B)**, bipolar cells **(C)**, rods **(D)**, cones **(E)**, S cones **(F)**, and Müller glia **(G)**.

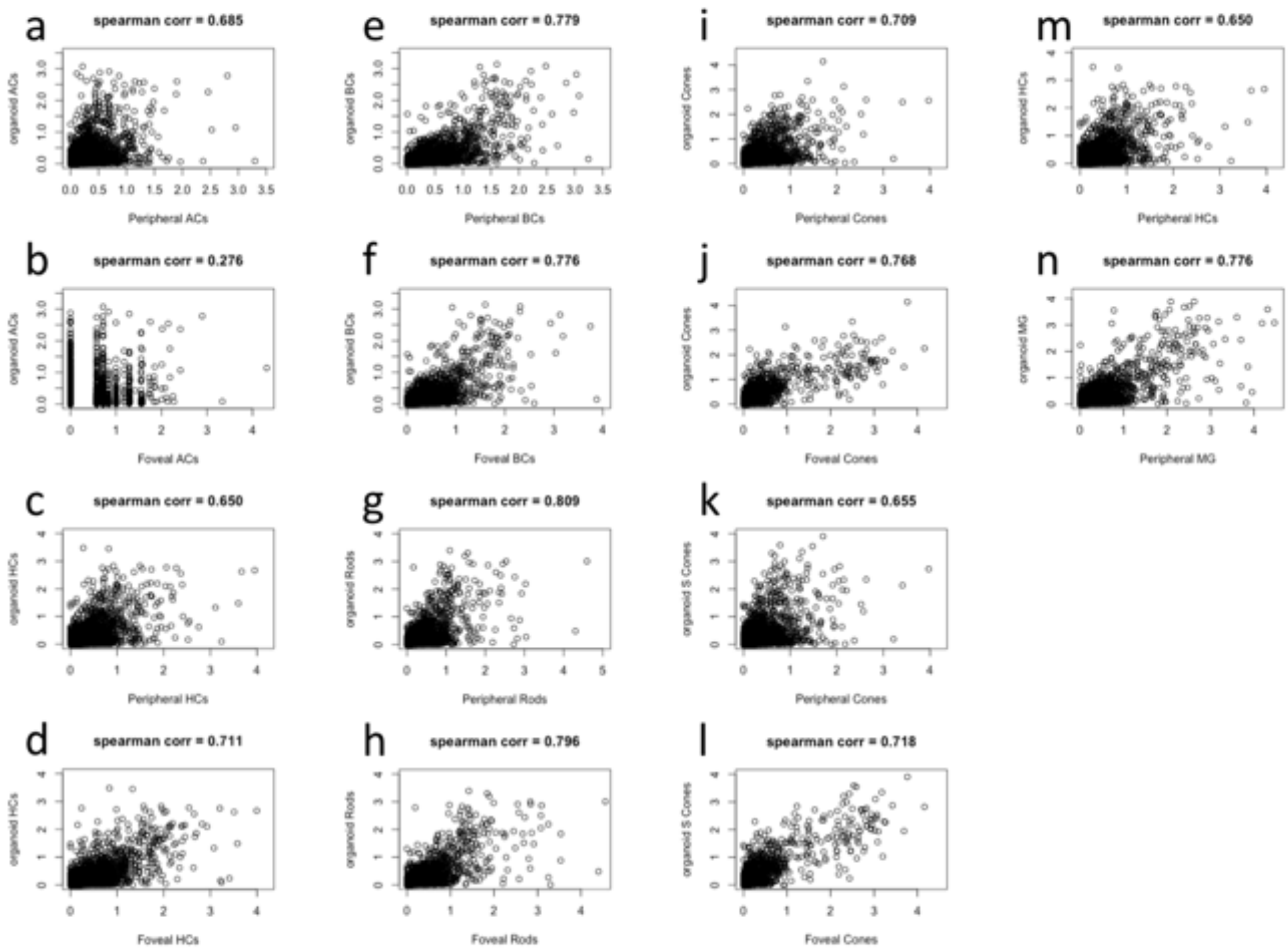

Supplementary Figure 11: Correlation of d170 retinal cell populations with adult peripheral and foveal retina scRNAseq data[24]. **A-N)** Scatter plots and Spearman correlations with peripheral **(A)** and foveal **(B)** amacrine cells, peripheral **(C)** and foveal **(D)** horizontal cells, peripheral **(E)** and foveal **(F)** bipolar cells, peripheral **(G)** and foveal **(H)** rods, peripheral **(I)** and foveal **(J)** cones, S-cones with peripheral **(K)** and foveal **(L)** cones, and peripheral **(M)** and foveal Müller glia **(N)**.

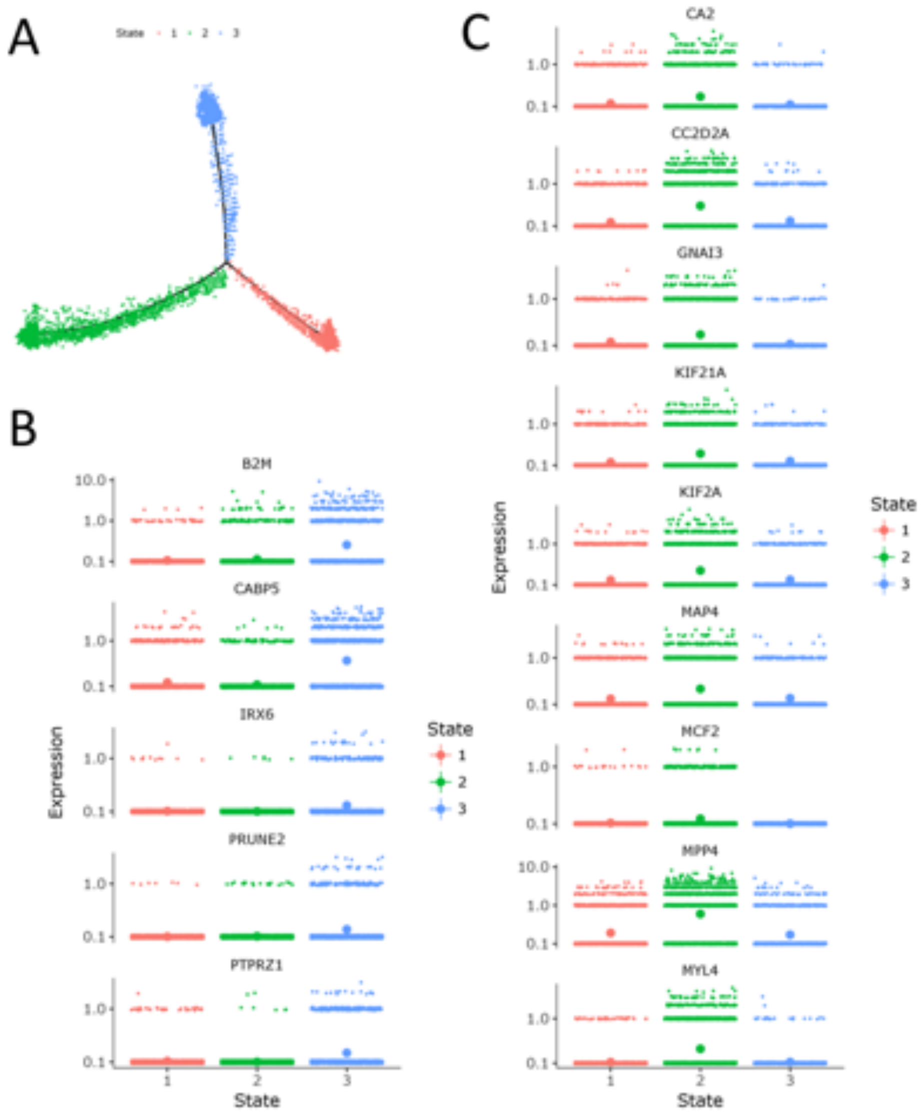

Supplementary Figure 12: Identification of novel rod- and cone-enriched genes from differential gene expression analysis at the node separating rods and cones. A) Trajectory of 5144 WT photoreceptors colored by state (state 2 = cones, state 3 = rods). **B-C)** Expression pattern of novel rod-enriched (**B**) and cone-enriched (**C**) genes, with the largest dot indicating average level of expression of each gene of interest.

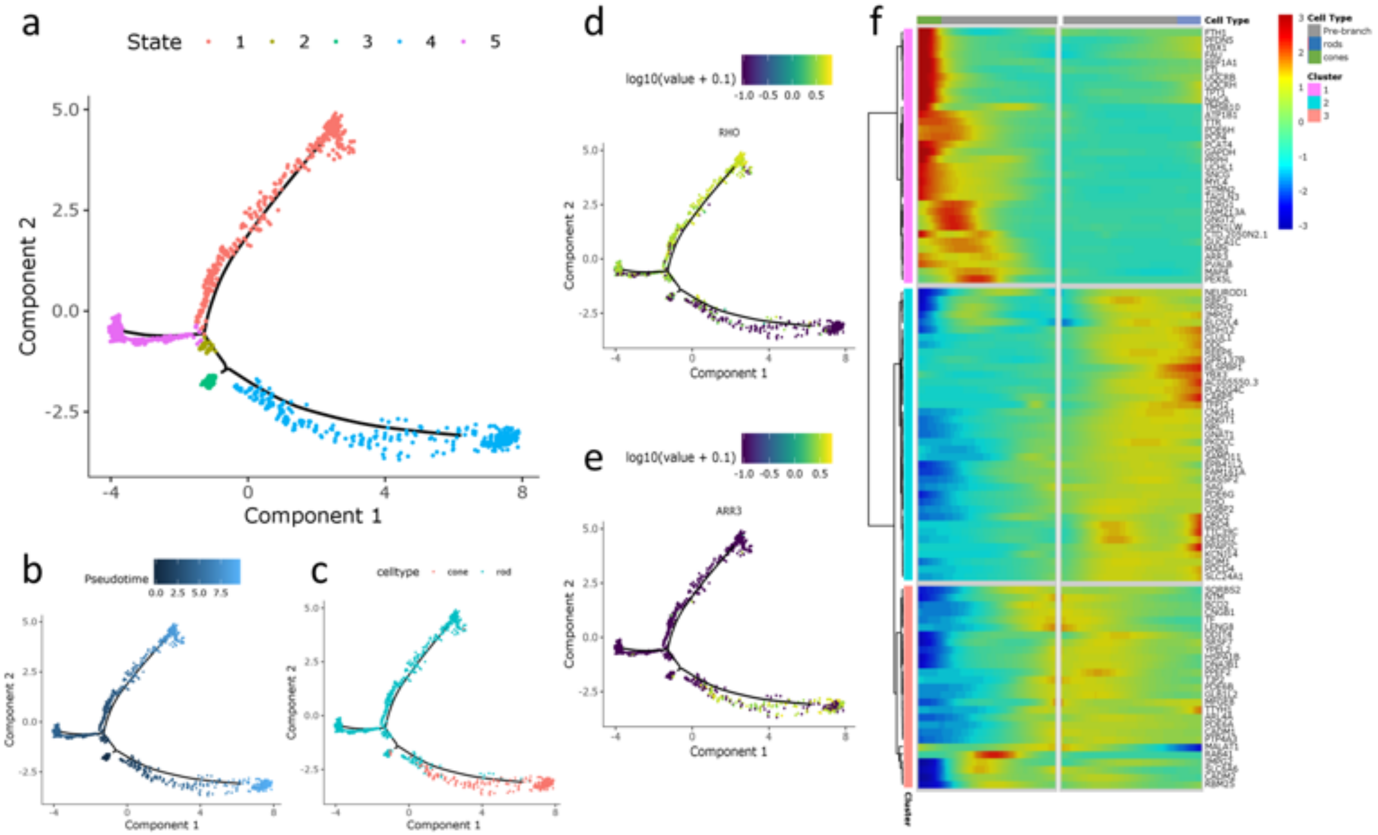

Supplementary Figure 13: Developmental trajectory of adult *in vivo* photoreceptors[24]. A-C) Trajectory colored by state **(A)**, pseudotime **(B)**, and cell type **(C)**. **D-E)** Expression patterns of RHO **(D)** and ARR3 **(E)** along the trajectory. **F)** Heatmap of the top 100 non-ribosomal differentially expressed genes between states 1 (rods) and 4 (cones).

A

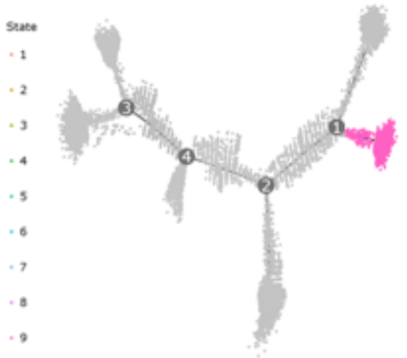

B

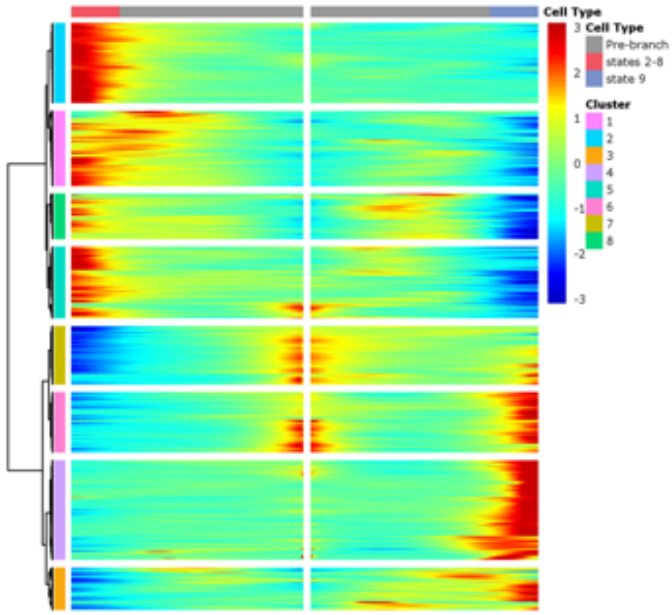

C

GO term	Fold change	p value
regulation of timing of cell differentiation	32.25	1.42E-02
protein refolding	22.57	8.06E-04
regulation of cellular response to heat	13.19	6.28E-04
oligodendrocyte differentiation	11.47	8.52E-03
cellular response to heat	10.12	1.97E-02
regulation of neural precursor proliferation	9.38	3.27E-02
endocrine system development	9.21	3.69E-02
cellular response to toxic substance	8.38	1.37E-02
response to reactive oxygen species	8.12	1.74E-02
response to unfolded protein	6.08	3.49E-03
mRNA splicing, via spliceosome	5.15	3.34E-02
epithelial cell differentiation	4.35	2.42E-03
gland development	4.28	1.35E-02
brain development	3.56	6.75E-05
sensory organ development	3.48	4.94E-02
generation of neurons	3.29	5.52E-03
negative regulation of cell differentiation	3.21	4.68E-02
negative regulation of transcription, DNA templated	2.6	3.93E-03
positive regulation of developmental process	2.53	1.52E-02
negative regulation of cell communication	2.44	2.77E-02

Supplementary Figure 14: Gene ontology enrichment analysis used to characterize the photoreceptors of state 9. A) Trajectory of combined WT and L75Pfs cells highlighting state 9 in pink. **B)** Heatmap of all genes differentially expressed at node 1, with genes hierarchically clustered. Clusters 3, 4, and 6 are highly enriched in state 9. **C)** GO term enrichment for genes in clusters 3, 4, and 6 (p values are Bonferroni corrected).

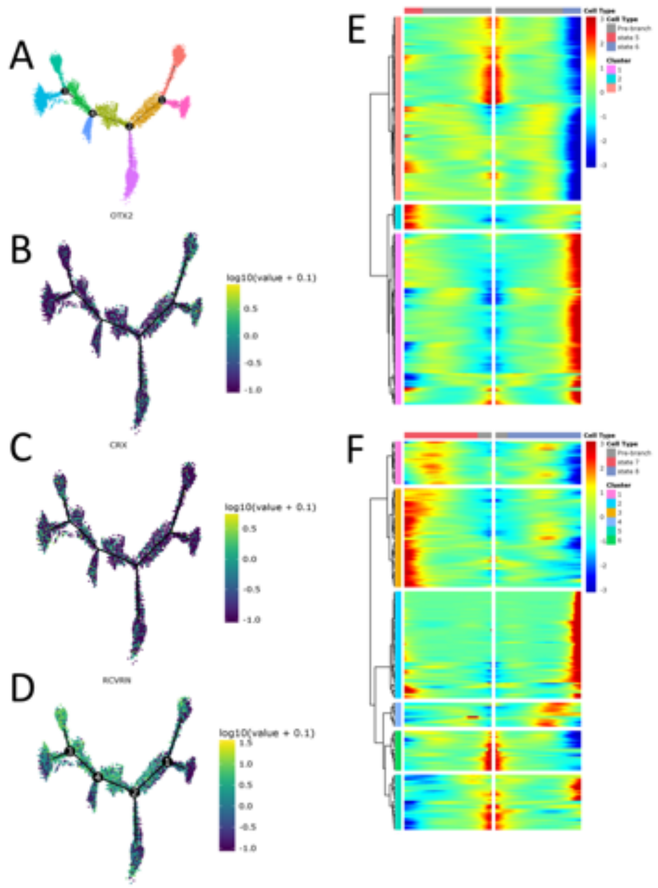

GO terms – State 5 enriched	fold enrichment	p value
Positive regulation of guanylate cyclase activity	>100	3.95E-03
mitochondrial coupled ATP synthesis coupled proton transport	38.4	2.25E-09
cristae formation	23.74	1.50E-08
mitochondrial electron transport, NADH to ubiquinone	23.46	3.82E-13
proteasomal ubiquitin independent protein catabolic process	21.86	1.06E-03
mitochondrial respiratory chain complex I assembly	17.08	6.94E-13
ATP hydrolysis coupled cation transmembrane transport	13.89	1.55E-05
aerobic respiration	9.32	5.91E-04
translation	3.54	3.65E-03
symbiont process	2.88	2.15E-02

GO terms – State 6 enriched	fold enrichment	p value
neural retina development	11.04	1.11E-02
photoreceptor cell differentiation	10.15	1.94E-02
brain development	3	1.61E-02
regulation of multicellular organismal process	1.92	2.10E-02

GO terms – State 7 enriched	fold enrichment	p value
positive regulation of guanylate cyclase activity	>100	4.31E-02
phototransduction	27.94	1.11E-02
neural retina development	26.24	1.31E-03
visual perception	7.68	6.15E-03

GO terms – State 8 enriched	fold enrichment	p value
rhodopsin mediated signaling pathway	>100	8.21E-07
negative regulation of inclusion body assembly	>100	3.14E-02
protein refolding	69.81	3.86E-03
platelet aggregation	66.49	4.59E-03
regulation of rhodopsin mediated signaling pathway	55.81	2.40E-05
chaperone cofactor dependent protein refolding	51.71	1.13E-02
eye photoreceptor cell development	37.89	2.83E-03
regulation of tumor necrosis factor mediated signaling pathway	35.8	4.31E-02
visual perception	20.74	6.90E-12
response to unfolded protein	15.18	1.96E-02
regulation of myeloid cell differentiation	15.18	1.96E-02
anatomical structure homeostasis	12.58	1.38E-03
leukocyte activation	6.61	4.07E-02
cellular response to organic substance	4.59	1.18E-04

Supplementary Figure 15: Gene expression and GO enrichment analyses used to identify developing photoreceptors and to characterize differences between the 2 cone states and the 2 rod states. A) Trajectory of combined WT and L75Pfs photoreceptors colored by state. **B-D)** Expression pattern of *OTX2* (**B**), *CRX* (**C**), and *RCVRN* (**D**) along the trajectory. **E)** Heatmap of genes differentially expressed between cone states 5 and 6 and their corresponding gene ontology enrichment based on gene clusters (gene cluster 1 is enriched in state 6; gene clusters 2 and 3 are enriched in state 5). **F)** Heatmap of genes differentially expressed between rod states 7 and 8 and their corresponding gene ontology enrichment based on gene clusters (gene clusters 1, 3, and 6 are enriched in state 7; gene clusters 2, 4, and 5 are enriched in state 8).

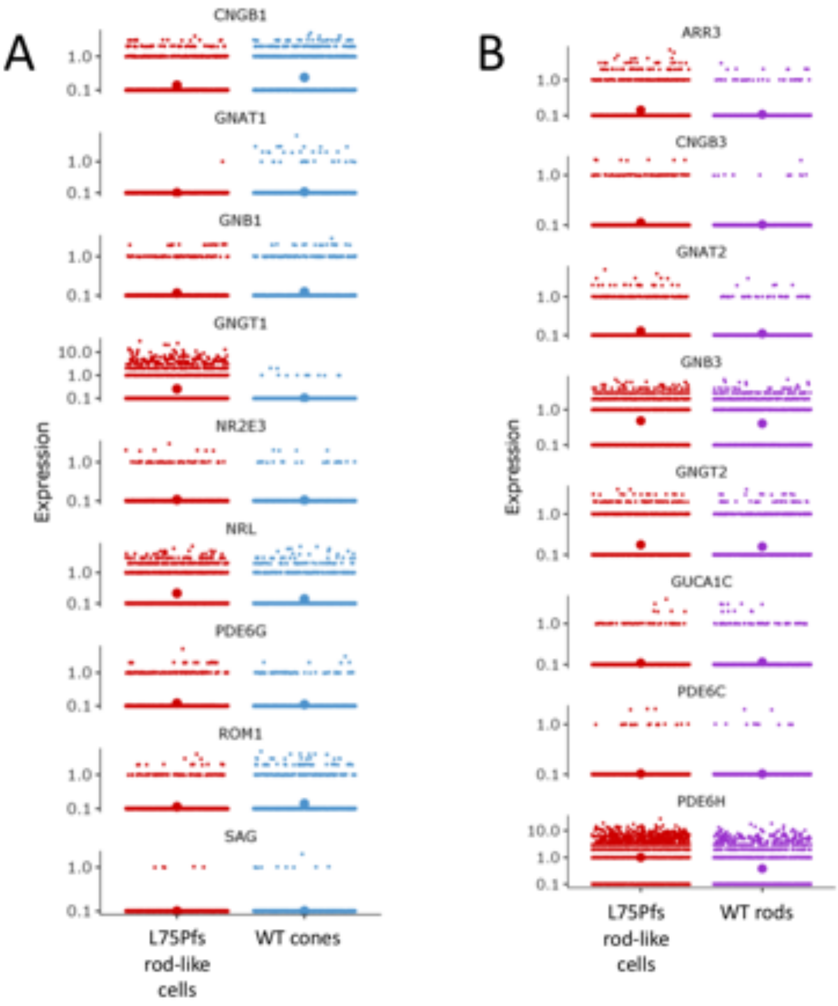

Supplementary Figure 16: Comparison of rod and cone genes in L75Pfs rod-like cells reveals low expression levels of rod or cone genes. A) Expression levels of rod genes in L75Pfs rod-like cells is comparable to WT cone expression levels of rod genes. **B)** Expression levels of most cone genes in L75Pfs rod-like cells is comparable to WT rod expression levels of cone genes. Exceptions include *ARR3* and *PDE6H.*

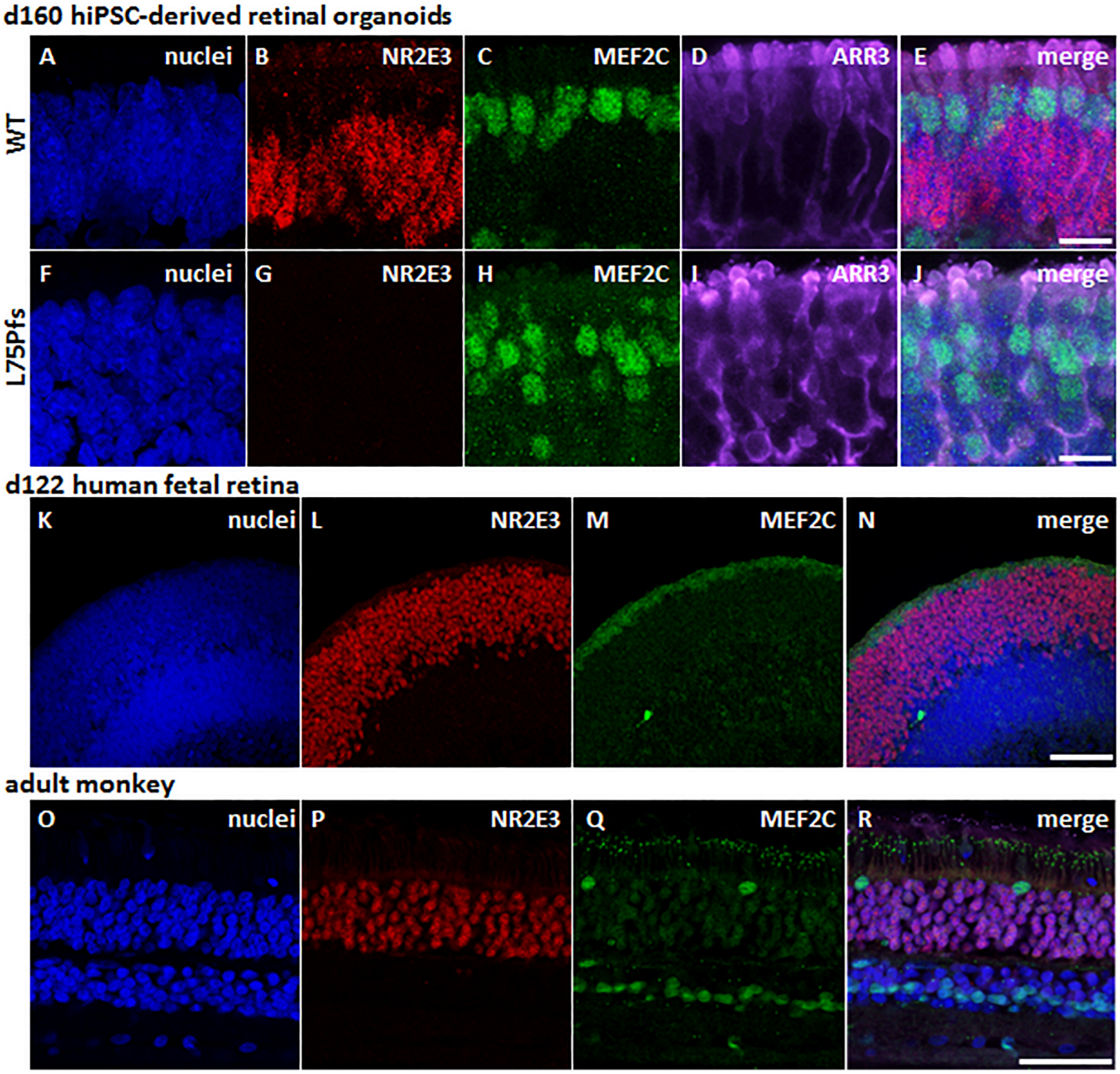

Supplementary Figure 17: Co-expression of MEF2C with ARR3, but not NR2E3, in hPSC-derived retinal organoids and human fetal retina supports a role for MEF2C in cone development. A-J) Confocal images of WT (**A-E**) or L75Pfs (**F-J**) d160 stage 3 (i.e., photoreceptor outer segment-containing) retinal organoids showing MEF2C (**C, H**) co-localization with ARR3 (**D, I**, merge in **E** and **J**), but not with NR2E3 in WT (**B**). **K-N)** Day 122 human fetal retina demonstrating localization of MEF2C (**M**) to the single layer of cone nuclei external to NR2E3+ developing rods (**L**, merge in **N**). **O-R)** Section from an adult monkey peripheral retina showing MEF2C (**Q**) localizing to two NR2E3-negative (**P**) nuclei in the ONL and a row of possibly Müller glia nuclei in the ONL (**Q**). Nuclei (blue) are shown in **A**, **F** and **K**. Scale bars: **A-E** and **F-J** = 10 microns. **K-N** and **O-R** = 50 microns.

Supplementary Tables

Supplementary Table 1: Marker genes used to call cluster identity

Cell type	Gene(s)
Rod photoreceptors	*NR2E3, NRL* (wild type cells only), *GNGT1, SAG*
Cone photoreceptors	*PDE6H, ARR3, OPN1MW, OPN1SW, GUCA1C , GNAT2*
Bipolar cells	*VSX1, VSX2, TMEM215, ISL1*
Photoreceptor/Bipolar precursors	*CRX, OTX2, PRDM1, VSX1*
Amacrine cells	*TFAP2A, GAD1, GAD2, CALB2*
Horizontal cells	*ONECUT1, ONECUT2, TFAP2B, PROX1*
Retinal ganglion cells	*SLC17A6, GAP43, NEFL, NEFM*
Muller glia	*SOX2, SOX9, VIM, CLU, DKK3*
Mitotic cells	*MKI67, TOP2A, NUSAP1, CENPF*

Supplementary Table 2: primary antibodies

ARR3	rabbit	LS Bio	1:100
ARR3	goat	Novus	1:100
CRALBP	mouse	Abcam	1:250
CRX	mouse	Abnova	1:1000
Ki67	rabbit	Abcam	1:100
G0α	mouse	Millipore	1:500
ML OPSIN	rabbit	Millipore	1:500
MEF2C	rabbit	Abnova	1:100
hNANOG	rabbit	Stemgent	1:100
NRL	goat	R&D Systems	1:300
NR2E3	mouse	Abcam	1:300
OCT3/4	mouse	Santa Cruz	1:100
PHALLOIDIN (f-actin)		Invitrogen	1:300
PKCα	rabbit	Sigma	1:50
RECOVERIN	rabbit	Millipore	1:2000
RHO (4D2 clone)	mouse	Millipore	1:100
SNCG	mouse	Abnova	1:500
S OPSIN	rabbit	Millipore	1:500
SOX2	goat	R&D Systems	1:500
VGLUT	guinea pig	Millipore	1:2000
VSX2	sheep	Exalpha	1:200

Supplementary Table 3: qPCR primers

β-actin	GCGAGAAGATGACCCAGATC<br>CCAGTGGTACGGCCAGAGG
CRX	TTTGCCAAGACCCAGTACCCAGA<br>TGCATTTAGCCCTCCGGTTCTTGA
GNAT1	ACGGGTACTCGCTGGAAGA<br>TCTCCGTACTGGATGTTGAGTG
NRL	CACGGTTCTCTGCATCGTTA<br>AAATTCGGGCATGACTTGAG
NR2E3	CCAGTCCCAAGTGATGCTGAG<br>GCGTTCCGCAGTGATAAACC
OPN1SW	TACGGCTTGTCACCATTCCT<br>GGATTCATCTGTCATGGCCT
RCVN	CTCCTTCCAGACGATGAAAACA<br>GCCAGTGTCCCCTCAATGAA
YWHAZ	ACTTTTGGTACATTGTGGCTTCAA<br>CCGCCAGGACAAACCAGTAT
