# Supplementary material for: Investigating cone photoreceptor development using patient-derived NRL null retinal organoids
Source: Commun Biol. 2020 Feb 21;3:82. doi: 10.1038/s42003-020-0808-5 (PMC7035245; doi:10.1038/s42003-020-0808-5)
Supplement: Supplementary file 3 — Description of additional supplementary items [file 42003_2020_808_MOESM3_ESM.docx]

**Supplementary Data 1: Genes differentially expressed between WT and L75Pfs cells at d100. Differential expression analysis was performed within each cluster and only genes with a statistically significant Bonferroni corrected p value and average natural log fold change > 0.5 were included. The “genotype” column indicates in which genotype each gene is enriched.**

**Supplementary Data 2: Genes differentially expressed between WT and L75Pfs cells at d170. Differential expression analysis was performed within each cluster and only genes with a statistically significant Bonferroni corrected p value and average natural log fold change > 0.5 were included. The “genotype” column indicates in which genotype each gene is enriched.**

**Supplementary Data 3: Ordering genes used for trajectory reconstruction.**

**Supplementary Data 4: Genes differentially expressed at the node separating WT rods and cones.**

**Supplementary Data 5: Genes differentially expressed between WT and L75Pfs cones.**

**Supplementary Data 6: Genes differentially expressed between WT rods and L75Pfs rod-like cells.**

**Supplementary Data 7: Genes differentially expressed between WT cones and L75Pfs rod-like cells.**

**Supplementary Data 8: Data input to create graphs for Figure 2O-Q**
